# Coupled jumping frogs/particle swarm optimization for estimating the parameters of three dimensional HIV model

**DOI:** 10.1186/1471-2334-12-S1-P82

**Published:** 2012-05-04

**Authors:** K Kamalanand, P Mannar Jawahar

**Affiliations:** 1 Madras Institute of Technology Campus, Anna University, Chennai, India

## Background

The three dimensional model of HIV/AIDS is the basis of active research since it includes most aspects regarding the interaction of HIV with patient’s immune system. This model is parameterized by six parameters namely the death rate of CD4 cells, rate of infection of CD4 cells by virus, death rate of CD8 cells, rate of increase of CD8 cells in response to increased viral load, rate of increase of viral load, and rate of decrease of viral load respectively. The objective of this work is to efficiently estimate the HIV parameters using available measurements of viral load.

## Methods

The HIV parameters were estimated using coupled jumping frogs/particle swarm optimization technique and viral load measurements. A total number of 25 particles and 25 frogs were used for estimation. The algorithm was run for 20 iterations. The performance of the proposed method for estimation of HIV parameters was assessed in terms of percentage accuracy and estimation time.

## Results

The percentage accuracy in estimation of HIV parameters a, b, c, d, e and f was found to be 93.30%, 92.40%, 94.04%, 93.01%, 100% and 98.41% respectively. The total time for estimation was observed to be 426 seconds. Further, the minimum estimation error was achieved within 12 iterations.

## Conclusion

For using HIV model for treatment planning, the model parameters must be estimated from measurements acquired on equipment which is accessible to local health services. Results demonstrate that the proposed method is efficient for estimation of all the six parameters of the HIV model.

